# Neural substrates of cue reactivity and craving in gambling disorder

**DOI:** 10.1038/tp.2016.256

**Published:** 2017-01-03

**Authors:** E H Limbrick-Oldfield, I Mick, R E Cocks, J McGonigle, S P Sharman, A P Goldstone, P R A Stokes, A Waldman, D Erritzoe, H Bowden-Jones, D Nutt, A Lingford-Hughes, L Clark

**Affiliations:** 1 Centre for Gambling Research at UBC, Department of Psychology, University of British Columbia, Vancouver, BC, Canada; 2 Department of Psychology, University of Cambridge, Cambridge, UK; 3 Centre for Neuropsychopharmacology, Division of Brain Sciences, Department of Medicine, Imperial College London, London, UK; 4 School of Psychology, College of Social Science, University of Lincoln, Lincoln, UK; 5 Computational, Cognitive and Clinical Neuroimaging Laboratory, Division of Brain Sciences, Imperial College London, London, UK; 6 Department of Psychological Medicine, Institute of Psychiatry, Psychology and Neuroscience, Centre for Affective Disorders, King’s College London, London, UK; 7 Division of Experimental Medicine, Department of Imaging, Imperial College London, London, UK; 8 National Problem Gambling Clinic, CNWL NHS Foundation Trust, Imperial College London, London, UK

## Abstract

Cue reactivity is an established procedure in addictions research for examining the subjective experience and neural basis of craving. This experiment sought to quantify cue-related brain responses in gambling disorder using personally tailored cues in conjunction with subjective craving, as well as a comparison with appetitive non-gambling stimuli. Participants with gambling disorder (*n*=19) attending treatment and 19 controls viewed personally tailored blocks of gambling-related cues, as well as neutral cues and highly appetitive (food) images during a functional magnetic resonance imaging (fMRI) scan performed ~2–3 h after a usual meal. fMRI analysis examined cue-related brain activity, cue-related changes in connectivity and associations with block-by-block craving ratings. Craving ratings in the participants with gambling disorder increased following gambling cues compared with non-gambling cues. fMRI analysis revealed group differences in left insula and anterior cingulate cortex, with the gambling disorder group showing greater reactivity to the gambling cues, but no differences to the food cues. In participants with gambling disorder, craving to gamble correlated positively with gambling cue-related activity in the bilateral insula and ventral striatum, and negatively with functional connectivity between the ventral striatum and the medial prefrontal cortex. Gambling cues, but not food cues, elicit increased brain responses in reward-related circuitry in individuals with gambling disorder (compared with controls), providing support for the incentive sensitization theory of addiction. Activity in the insula co-varied with craving intensity, and may be a target for interventions.

## Introduction

Pathological gambling (now termed gambling disorder) is the first behavioral addiction to be recognized in the substance-related and addictive disorders section of the DSM-5. Within this section, craving was introduced as a criterion for the substance-use disorder diagnoses, largely based on its value as a biomarker and predictor of outcomes.^1^ Craving is not currently listed as a criterion for gambling disorder, despite the centrality of this feature to the development and maintenance of the disorder,^2^ and as a predictor of relapse^3^ and treatment attrition.^4^

In addictions research, craving is widely studied using the cue reactivity procedure, in which participants are exposed to Pavlovian-conditioned stimuli that are reliably paired with substance use, such as images of lit cigarettes or hypodermic needles. The incentive sensitization theory of addiction^5, 6^ posits that dopaminergic reward circuitry is activated by such cues, and thus the cue reactivity response in this circuitry is hypothesized to be increased in groups with substance-use disorders compared with controls. A meta-analysis of cue reactivity in alcohol use disorders found increased reactivity in posterior cingulate cortex, precuneus and superior temporal gyrus,^7^ suggesting these regions, although not typically considered part of the reward network, may indeed be sensitized. More recent studies comparing patients with alcohol use disorders with controls have observed increased reactivity within the reward network, including the orbitofrontal cortex,^8, 9^ anterior cingulate cortex (ACC) and ventromedial prefrontal cortex (PFC).^9^ In addition, correlations with craving in substance-use disorders have been observed in the bilateral insula^10^ and ventral striatum.^7^

Cue reactivity can also elicit craving responses in individuals with gambling disorder, as measured by self-report scales and physiological responses.^10, 11^ However, past experiments assessing the neural substrates of this cue reactivity with neuroimaging have revealed mixed results. An early study using auditory descriptions of gambling scenarios reported a decreased response in reward-related circuitry (ACC and caudate).^11^ Three subsequent studies found effects in the opposite direction, with an increased response to gambling videos in the dorsolateral PFC, parahippocampal gyrus and occipital cortex,^12^ increased response to gambling images in occipito-temporal regions, posterior cingulate cortex, parahippocampal gyrus and amygdala,^13^ and increased response in the medial PFC.^14^ In the latter study, cue reactivity to gambling cues in individuals with gambling disorder was compared with the reactivity to cocaine cues in individuals with cocaine dependence. The same region of the medial PFC was activated by cues in the gamblers and the cocaine-dependent group. One of the above studies looked for correlations with subjective cravings, finding that ratings taken after the scan predicted greater signal change to gambling cues in anterior insula, PFC and caudate.^13^ Although not traditionally considered as part of the reward network, the insula may have a key role here, as lesions to this region have been associated with an abolished urge to smoke^15^ and reduced nicotine withdrawal,^16^ as well as with attenuated gambling-related cognitive distortions.^17^ A direct connection between the anterior insula and the ventral striatum has recently been established,^18^ providing a pathway for integrating insula processing with the reward network.

One challenge with capturing cue reactivity in gambling disorder, and a potential reason for the mixed neuroimaging results untill now, is the greater range and specificity of associated cues compared with substance-use disorders. As an example, presentation of lottery cues to regular horse-race gamblers drove only modest changes in craving, relative to cues associated with the preferred activity.^19^ In the present study, we created a culturally appropriate image set and selected cues that were relevant to each participant. We predicted that such cues would elicit craving in a group with gambling disorder, as well as increased neural activity in reward-related circuitry, relative to control participants. Within individuals with gambling disorder, we further hypothesized that insula activity would correlate positively with craving ratings obtained during the task.

In addition to looking for relative increases or decreases in activity, we analyzed functional connectivity patterns for the nucleus accumbens, a region of the ventral striatum that receives dopaminergic inputs from the midbrain and has bidirectional connections with prefrontal cortex.^20^ Impaired connectivity between this region and the dorsolateral PFC was previously associated with craving in patients with alcohol use disorders,^21^ and this circuit is implicated in the cognitive control of craving in smokers.^22^ We predicted that regions of the PFC would show decreased connectivity with the nucleus accumbens in the group with gambling disorder, and that within the patient group, craving would be associated with reduced connectivity between nucleus accumbens and the PFC.

Our cue reactivity procedure also included a set of high-caloric sweet food images as a ‘natural’ reward. There is considerable overlap in the neural response to food cues and drug cues. A meta-analysis comparing the neural response to smoking cues and food cues found overlapping activation of the striatum, insula and orbitofrontal cortex.^10^ More recently, overlapping reactivity has been observed for multiple types of drug cues, natural rewards (including food and sex) and gambling. Regions responsive to all cues include the striatum, the anterior cingulate and the insula.^23^ Neural responsivity to food cues is modulated by multiple factors including motivational state and food-cue palatability.^24^ It is not yet known if food-cue reactivity is altered in gambling disorder. The reward-deficiency hypothesis of addiction vulnerability posits a reduction in reward-related activity across multiple types of rewards.^25, 26^ Recent experiments have indicated blunted sensitivity to ‘natural’ rewards in gambling disorder^27^ and nicotine dependence,^28^ in line with reward deficiency, and we sought to corroborate these findings in our own data, using highly appetitive food cues.^29^

## Materials and methods

### Participants

Individuals with gambling disorder (*n*=20, all male) were recruited from the National Problem Gambling Clinic, London, and healthy controls (*n*=22, all male) were recruited through community advertisements. Sample size was informed by power calculations. Between-group comparisons in groups with 20 participants have a power of 0.80 to detect an effect size of ~0.9, which is a plausible effect size based on previous literature.^30^ Gambling disorder was confirmed using DSM-IV criteria (for pathological gambling) and corroborated by scores ⩾8 on the Problem Gambling Severity Index (PGSI).^31^ See Supplementary Information 1 for full inclusion criteria. Patients were scanned while awaiting or undergoing psychological treatment for gambling disorder, and had been abstinent from gambling for a median of 31 days (range=2–120 days) before testing. All but one control participant scored zero on the PGSI, with this participant scoring two. The UK National Research Ethics Committee approved the protocol, and all volunteers provided written informed consent. These data were collected as part of a larger study including multiple functional magnetic resonance imaging (fMRI) tasks and positron emission tomography scans.^32^ One gambling disorder and two control participants were excluded due to excess motion during the functional scans. Excess motion was defined using two criteria: first, a maximum frame-wise displacement >4 mm and second, more than 10% of volumes identified as containing extreme intensity difference values by the dvars metric of FSLMotionOutliers. These criteria were established before the analysis. This combination of criteria ensured that participants with just one large movement would not be excluded. Of those included, only two participants had a maximum frame-wise displacement >2 mm, and all were <3 mm. One additional control was excluded because of incomplete data. Thus, our analyses include data from 19 individuals with gambling disorder and 19 controls.

Participants completed the Beck depression inventory (BDI-II),^33^ the Spielberger state-trait anxiety index (STAI),^34^ the Fagerstrom test for nicotine dependence (FTND)^35^ and the alcohol use disorders identification test (AUDIT).^36^ Current and lifetime psychiatric disorders were assessed using the mini international neuropsychiatric interview (MINI-5)^37^ administered by a psychiatrist. As a result of this interview, no participants were diagnosed with a current psychiatric illness (excluding gambling disorder) in line with our inclusion criteria. Intelligence quotient (IQ) was assessed using the vocabulary and matrix reasoning subtests of the Wechsler abbreviated scale of intelligence.^38^ Potential disordered eating behavior was measured using the Eating disorder examination questionnaire,^39^ Dutch eating behaviour questionnaire^40^ and three factor eating questionnaire^41^ with a focus on the restraint subscales, which we have seen to predict reward network activity during food picture evaluation (Goldstone, unpublished data).

### Procedures

**Figure 1:**
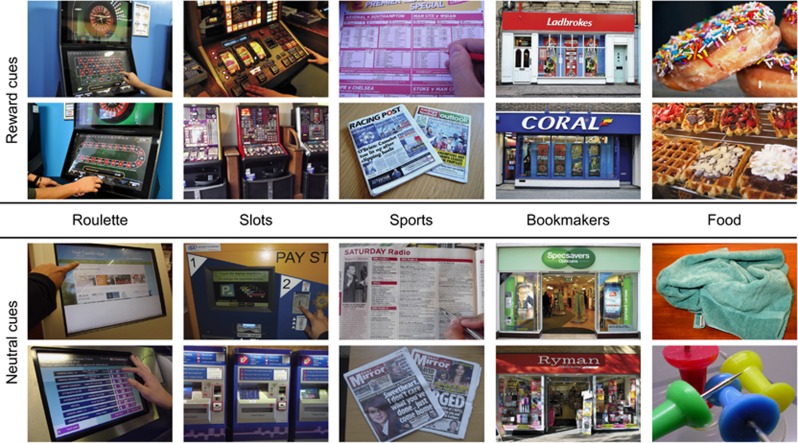
Examples of the cues used in the task.

Brain images were acquired on a Siemens 3T scanner (Supplementary Information 2). Four categories of photographs were shown to participants during the MRI scan: gambling cues, gambling-matched neutral cues, food cues, and food-matched neutral cues (Figure 1). There were four subtypes of gambling cues: photographs of the shop-fronts of bookmakers from the UK high street, as well as ‘action’ images from the three most common preferred forms of gambling among our clients: electronic roulette, sports betting and slot machines. For each participant, we selected the two forms most relevant to their personal game preferences, as well as the shop-fronts. The ubiquity of betting shops in the UK that offer multiple forms of gambling (including electronic gaming machines and sports betting) means that shop-fronts themselves may be powerful cues, as shown for branded gambling advertisements.^42^ Fourteen patients were shown the roulette and sports images, and five were shown the roulette and slot machine images. The task design was matched for the controls as closely as possible (15 roulette and sports, 4 roulette and slot machines). Gambling-matched neutral cues were selected in a pairwise manner to the gambling cues, based on the presence of faces, hands, actions, electronic devices, touch screens and overall composition. Images of bookmaker shop-fronts were paired with shop-fronts that had no associations with gambling. Neutral cues matched to the food cues included pictures of objects such as furniture and clothing.

Both the gambling cues and their matched neutral cues were either taken locally by the experimenters or purchased from a stock image company. Food cues were close ups of sweet foods used in previous fMRI studies.^29, 43^ To control for the (known) impact on fasting on neural responses to food,^29, 43^ and the potential impact of fasting on neural response to gambling cues, we instructed participants to eat a light meal ~2 h before the scan (Table 1). The cue reactivity task commenced between 10:44 hours and 15:50 hours in the control participants, and between 11:16 hours and 15:53 hours for the gambling disorder group. Participants were allowed to smoke nicotine on the day of the test session, up to two hours before the start of the task.

Stimuli were presented in a blocked design (Supplementary Information 3). Each block contained five images from the same category, presented for 4.8 s per image. To maintain attention, participants were asked to press a button with each new image. Three control participants did not adhere to this instruction, but all results remain qualitatively unchanged in sensitivity analyses excluding these three participants (Supplementary Information 4). Rest blocks consisted of a fixation cross for 24 s. At the end of each block, participants gave a craving rating (‘I crave gambling right now’) ^44^ from strongly disagree (one) to strongly agree (nine), with a 5 s time limit. Thus, participants provided gambling craving ratings after each cue condition and rest block, to assess if craving was specific to the gambling cues or generalized across conditions. We also asked participants to rate their craving to gamble before they entered the scanner, to establish a baseline craving score. Participants were instructed to imagine that they were in the place pictured in each photograph or interacting with the item shown. There were two runs of the task, each lasting 7.5 min.

### Data analysis

Demographic and clinical characteristics were analyzed using R (R core Team, Vienna, Austria) using unpaired *t*-tests or Wilcoxon rank-sum tests (two-tailed). Analyses of the craving ratings were carried out using two mixed design ANOVAs. The first tested for any difference in the ratings after the three types of non-gambling cues. The second tested for differences after gambling cues, non-gambling cues and rest. Greenhouse-Geisser correction was applied to all ANOVA within-subject contrasts where the assumption of sphericity had been violated. For those data where the assumption of normality had been violated, a robust ANOVA using trimmed means was carried out.^45^ For all models, the robust ANOVA revealed qualitatively the same results and so are not reported here. To establish if craving within the patients with gambling disorder was elevated throughout the task, baseline craving ratings taken before the scan were compared with rest block craving ratings within the task using a Wilcoxon signed-rank test (two-tailed).

fMRI data were analysed using the FMRIB Software Library (FSL). See Supplementary Information 5 for full details of the pre-processing. Two statistical analyses were carried out using a general linear model (GLM) approach in FEAT (FSL Expert Analysis Tool). The first was an activity analysis and the second a psychophysical interaction (PPI) functional connectivity analysis. For the activity analysis, a model of the experimental events was constructed by convolving the onset and duration of the cue blocks with a gamma haemodynamic response function (with a time-to-peak of 6 s) at the individual subject level. A single boxcar regressor was created for each cue type (gambling, neutral, food, food-matched neutral). Six standard motion regressors were also entered into the model. FSL Motion Outliers was used to identify volumes with large intensity changes remaining after motion correction using the dvars metric.^46^ A single regressor for each identified volume was also entered into the model on a subject-by-subject basis. Four contrasts were specified: gambling cues>gambling-matched neutral cues, food cues>food-matched neutral cues, gambling-matched neutral cues>gambling cues and food-matched neutral cues>food cues. Note that, while we collapsed across the non-gambling cue types for the analysis of the behavioral ratings, we did not collapse across the non-gambling cues for the imaging analysis. For the functional connectivity analysis, the GLM was expanded to include PPI regressors. The bilateral nucleus accumbens seed region was defined using FSL FIRST at the individual subject level on the T1-weighted structural scans. A mean time course from this seed region was extracted for each run for each subject, and entered as a physiological regressor in the GLM. PPI regressors were then created by multiplying the (demeaned) physiological regressor with the task regressors. A PPI term was included for each condition, but only the gambling-related contrasts were included in this model.

For each GLM, the results of the two runs for each participant were combined using a fixed effects model. Group statistics were then carried out using FLAME (FMRIB’s local analysis of mixed effects). For each of the lower-level contrasts, the mean result of the gambling disorder group was inspected, and two contrasts were carried out to examine (unpaired) group differences (gambling disorder>controls and controls>gambling disorder). Whole-brain group-level statistics were corrected for family-wise error using cluster-based thresholding (*Z*=2.3, *P*<0.05). For each significant cluster of activity, the peak voxel is reported in MNI coordinates (*x*, *y*, *z*). For the functional connectivity analysis, a pre-threshold mask was used to restrict the analysis to the prefrontal cortex, the insula and the striatum (covering 553 632 mm^3^) as our *a priori* regions of interest concerned connectivity between the nucleus accumbens and these regions. For any observed group differences, FSLs Featquery was used to interrogate the direction of the effects. For this, the median percent signal change within the cluster was calculated at the first level of the GLM for each participant.

In both the activity and connectivity analyses, we explored individual differences within the gambling disorder group as a function of the mean craving rating (following the gambling blocks), gambling severity (PGSI) and number of days abstinent. We also included the clinical measures that differed between our groups (see ‘Results’ section) to ensure any observed group differences were not explained by these differences. All individual difference measures were demeaned and included as covariates in a group-level analysis.

## Results

### Group characteristics

The groups did not differ significantly on age, IQ or dietary restraint scores. Participants with gambling disorder scored significantly higher on the BDI-II, STAI and AUDIT (Table 1), but did not meet criteria for current depression, anxiety or alcohol dependence. The gambling disorder group also scored higher on body mass index, with two of the patients scoring over 30. All fMRI contrasts using the food cues were unchanged with the removal of these two participants. The two groups were matched for number of smokers, but of the participants who did smoke, the gambling disorder group scored higher than the control participants on the FTND.

### Craving ratings

**Figure 2:**
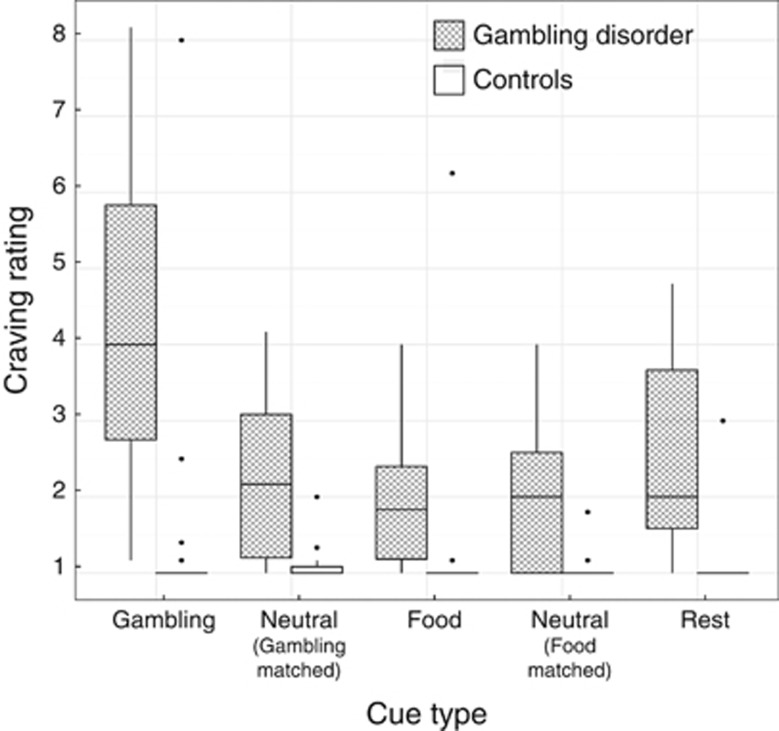
Craving to gamble ratings during the functional magnetic resonance imaging task. Ratings were provided after each block using a nine point Likert scale. The median and inter-quartile range (IQR) are represented by the boxplot. The whiskers extend to the minimum/maximum scores within 1.5 times the IQR, and the dots are individual scores that fall outside of this range.

Figure 2 shows the craving ratings after each experimental condition. We first confirmed that there was no significant difference between the craving ratings following the three non-gambling cue types, main effect: F(1.45, 52.31)=0.81, *P*=0.42, Cue type by Group interaction: F(1.45, 52.31)=2.34, *P*=0.12. Therefore, we averaged these ratings for the non-gambling blocks for the omnibus ANOVA of context (gambling cues, non-gambling cues, rest) by group. This analysis revealed a significant context × group interaction (F(1.25, 45.01)=11.06, *P*<0.001), driven by increased craving in the gambling disorder group following gambling cues relative to both neutral cues, F(1, 36)=24.38, *P*<0.001 and rest blocks, F(1, 36)=20.47, *P*<0.001, as well as significant main effects of group (gambling disorder>controls, F(1, 36)=23.56, *P*<0.001) and context, F(1.25, 45.01)=21.15, *P*<0.001. Within the patient group, craving ratings did not increase significantly from the pre-scan baseline (median=2) to the resting blocks (median=2.33, *P*=0.48, *r*=0.16).

### fMRI: cue reactivity

#### Activity analysis

**Figure 3:**
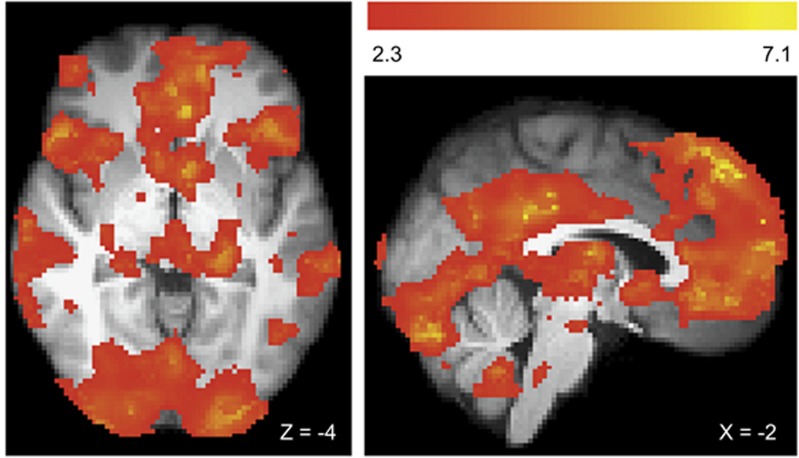
Cue-related activity to gambling>gambling-matched neutral contrast in the gambling disorder group. We observed three clusters of activity that showed a relative activity increase for gambling cues compared with gambling-matched neutral cues. An extensive cluster (covering 57 425 voxels in 2 mm standard space) extended to multiple brain regions, and so we report local maxima (*Z*>6.5). Peaks were localized to the left (−2, −42, 28, *Z*=7.14) and right (−4, −32, 32, *Z*=6.75) posterior cingulate gyrus, the left superior frontal gyrus (−2, 46, 50, *Z*=7.08), the left frontal pole ((−4, 50, 46, *Z*=7.01) and (−4, 58, 4, *Z*=6.81)) and the left paracingulate gyrus (−12, 50, 12, *Z*=6.54). Two smaller clusters showed peaks within the left angular gyrus (−52, −52, 40, *Z*=6.33) and the right lateral occipital cortex (62, −58, 34, *Z*=4.09). All images cluster corrected, *Z*>2.3, *P*<0.05 and presented using radiological convention.

The contrast of gambling>gambling-matched neutral cues revealed three clusters of activity within the gambling disorder group (Figure 3). A large cluster had local maxima within the left posterior cingulate gyrus, the left superior frontal gyrus, the left frontal pole and extended to multiple regions including the bilateral ventral striatum and medial PFC. Two additional clusters were observed with peaks within the left angular gyrus and right lateral occipital cortex. See Supplementary Figure 1 for the results of this contrast within the control group.

**Figure 4:**
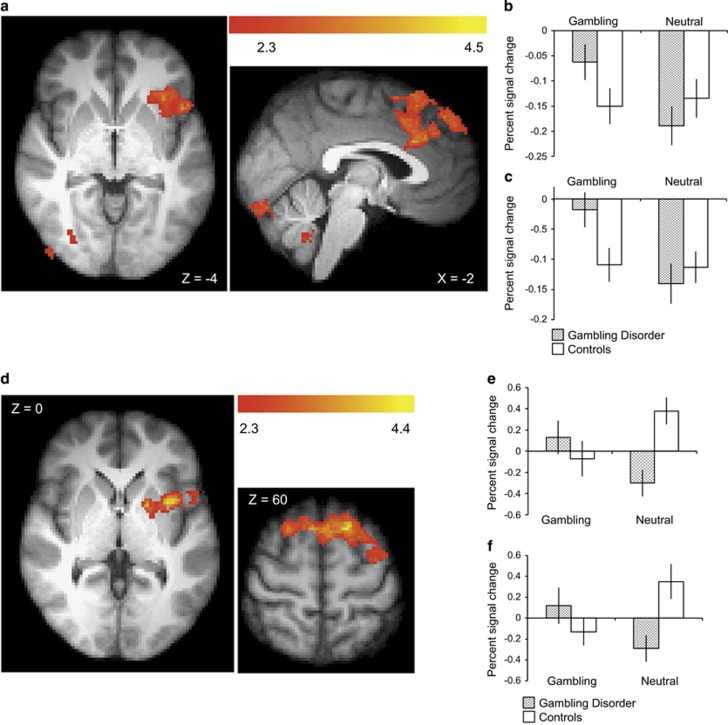
Group differences in gambling cue reactivity. Top panel: (**a**) Activity differences. The gambling disorder group showed increased activity relative to controls, in the gambling>gambling-matched-neutral contrast, in four clusters. One peaked within the anterior cingulate cortex (−2, 22, 28, *Z*=3.85), extending to the superior frontal gyrus. One peaked in the left frontal operculum (−48, 16, −4, *Z*=3.56) extending to the left insula, one peaked in the right inferior frontal gyrus (52, −52, −26, *Z*=4.33). An additional cluster was observed in the cerebellum (−16, −48, −42, *Z*=4.45), (**b**) extracted signal from the operculum/insula cluster, (**c**) extracted signal from the anterior cingulate cluster. Bottom panel: (**d**) Functional connectivity differences. The gambling disorder group showed increased connectivity changes, in the gambling>gambling-matched neutral contrast, between the nucleus accumbens and two clusters; one peaked within the left insula (−34, 6, 0, *Z*=4.07) (**e**), and the second within the superior frontal gyrus (−18, 18, 60, *Z*=4.35) (**f**).

Compared with controls, individuals with gambling disorder showed increased activity to gambling>gambling-matched neutral cues in four clusters, including left insula/frontal operculum and ACC/superior frontal gyrus (Figures 4a–c). Inspection of the extracted signal from these regions revealed that, compared with the implicit rest baseline, these regions showed a decrease in activity during neutral blocks in both groups of participants, but during gambling blocks only the control participants showed this reduction.

In the gambling disorder group, the contrast of food>food-matched neutral cues revealed two clusters of activity in the occipital pole and the right insula (Supplementary Figure 2). We observed no significant group differences between gambling disorder and control groups in the food>food-matched neutral cue contrast. In light of the modest food-cue reactivity responses observed within the gambling disorder group, and the absence of a significant group difference, a follow-up analysis was performed to further test whether we had elicited food-cue reactivity. We combined gambling disorder and control participants to look for an overall appetitive response to the food stimuli. This identified significant clusters in occipital cortex (−8, −96, 2, *Z*=9.15), paracingulate gyrus (−14, 48, 4, *Z*=5.27), insula (38, 8, −14, *Z*=4.84), ACC (−2, −14, 30, *Z*=3.77) and deactivations (that is, food<neutral) in left (42, −72, 20, *Z*=5.68) and right (42, −72, 20, *Z*=5.68) lateral occipital cortex (Supplementary Figure 3).

#### Connectivity analysis

In the gambling disorder group, the contrast of gambling>gambling-matched neutral cues revealed increased functional connectivity between the nucleus accumbens and the right inferior frontal gyrus (52, 24, 2, *Z*=3.97) (Supplementary Figure 4). In the direct group comparison, the participants with gambling disorder showed increased functional connectivity compared with controls, between the nucleus accumbens and two clusters: left insula cortex extending to left putamen, and superior frontal gyrus (Figures 4d–f). Inspection of the extracted signal from these regions revealed this effect was driven by the gambling disorder group showing relatively decreased connectivity during the neutral blocks, and controls showing relatively increased connectivity.

### fMRI: clinical correlations

To ensure our group differences were not driven by the clinical measures that differed between our groups we considered entering BDI-II, STAI, AUDIT and FTND into this analysis. We could not include FTND due to the small proportion of those participants who were smokers. BDI-II and STAI were strongly correlated (Supplementary Table 1), and so, of these measures, only BDI-II was entered. Similarly, a significant negative correlation was observed between craving ratings and days abstinent (*r*=−0.53, *N*=19, *P*=0.019); whereas this provides external validity to our cravings measure, it precluded the further inclusion of length of abstinence as a correlated regressor. We therefore tested for neuroimaging correlations with the following clinical variables in the gambling disorder group: craving scores, BDI-II, AUDIT and PGSI.

#### Activity analysis

**Figure 5:**
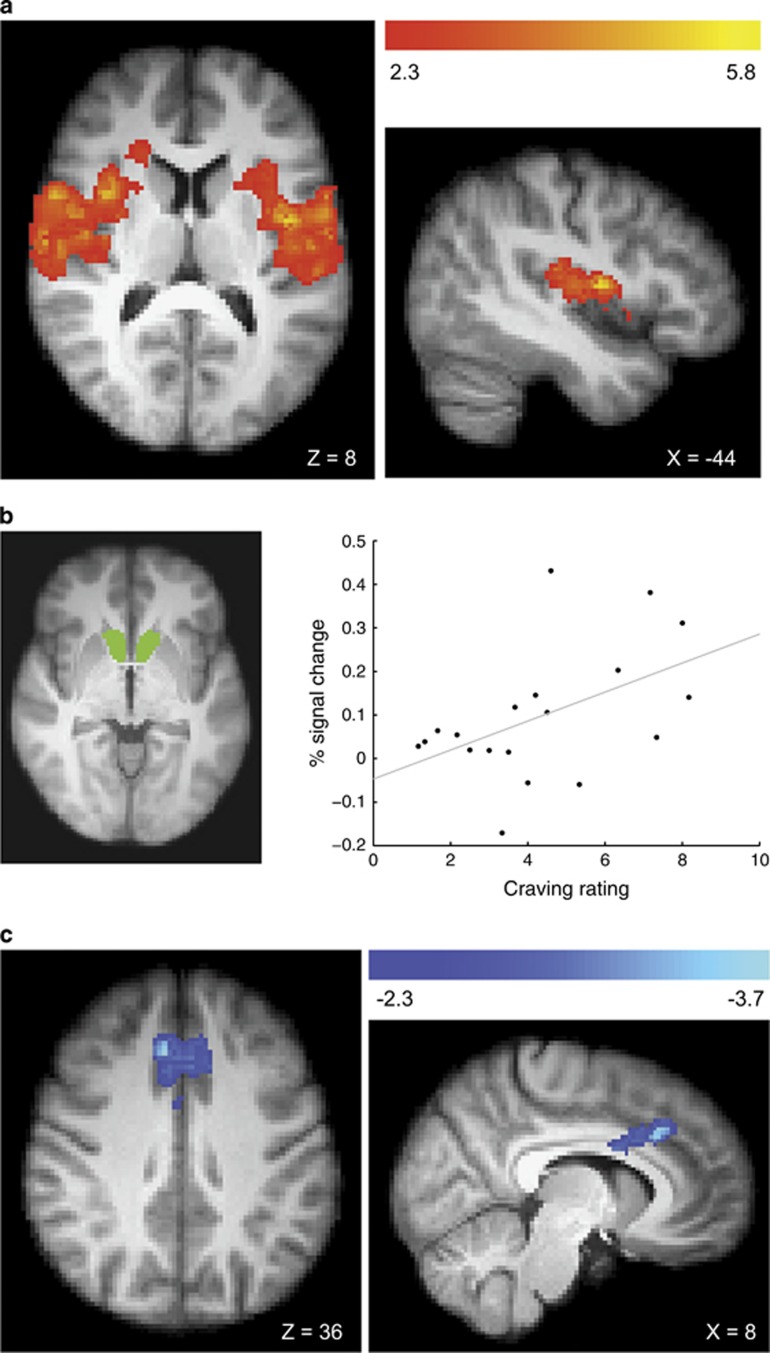
Correlations between craving ratings and gambling cue reactivity (gambling>neutral contrast) within the gambling disorder group. The whole-brain activity analysis (**a**) revealed three clusters showing a positive correlation, one peaked within the right insula (38, 4, 8, *Z*=5.84), a second within left central operculum (−44, −4, 10, *Z*=5.32) extending to the left insula and a third in the cerebellum (−10, −40, −10, *Z*=3.82). A region-of-interest analysis of activity within the bilateral nucleus accumbens (**b**) revealed a positive correlation between craving ratings and the percent signal change within this mask (*r*(19)=0.491,=*P*<0.05). The bilateral nucleus accumbens shown here in green, as defined by the Harvard–Oxford subcortical structural atlas. The functional connectivity analysis (**c**) revealed a single cluster showing a negative correlation with a peak within the paracingulate gyrus (2, 24, 36, *Z*=3.72).

Mean craving ratings in the gambling disorder group predicted greater activity for gambling>gambling-matched neutral cues in three clusters: the right insula, the left central operculum/ left insula and the cerebellum (Figure 5a). Higher BDI-II scores also predicted greater activity to gambling>gambling-matched neutral cues in left frontal pole (−2, 70, 12, *Z*=4.89), left postcentral gyrus (−66, −20, 26, *Z*=4.63) and cerebellum (−38, −70, −22, *Z*=3.60). Several cue reactivity studies in substance-use disorders have reported a correlation between subjective craving and activity in the ventral striatum.^7^ In a supplementary test using the nucleus accumbens region-of-interest (as defined for the connectivity analysis), a significant correlation between the signal change in the gambling>gambling-matched neutral cues contrast and craving ratings was observed in the gambling disorder group (Figure 5b). No regions showed any correlations with AUDIT or PGSI scores.

#### Connectivity analysis

For the functional connectivity analysis, higher craving ratings were associated with reduced connectivity between nucleus accumbens and medial PFC (Figure 5c). Higher BDI-II scores predicted reduced connectivity between the nucleus accumbens and the left precentral gyrus (−40, 0, 40, *Z*=4.01) and the left caudate (−16, 18, 8, *Z*=4.00). No regions showed any correlations with AUDIT or PGSI scores.

## Discussion

The present study examined the neural basis of cue reactivity in a group of patients attending treatment for gambling disorder. The most commonly reported problematic forms of gambling were electronic roulette and sports gambling, and our gambling cues were individually tailored to game preferences. Ratings taken on a block-by-block basis during scanning confirmed that craving was reliably induced by our gambling cues, and these cravings were specific to both cue type (relative to neutral, food and rest blocks) and the participants with gambling disorder. The gambling blocks in our task elicited strong whole-brain level activations across subcortical, limbic and cortical (occipital, temporal, frontal) networks. Our gambling disorder group showed specific increases (group × cue interactions) in this neural reactivity in anterior cingulate and insula.

Within the gambling disorder group, activity in bilateral insula (whole brain) and nucleus accumbens (region-of-interest) was significantly associated with craving. As the mean level of craving increased, the reactivity of these regions to gambling cues (relative to neutral cues) increased. Within the insula, this activity was localized to the mid and posterior regions, which have been associated with primary interoception, as opposed to anterior insula, which is implicated in higher-level aspects of interoceptive awareness.^47, 48^ In addition, the gambling disorder group showed increased connectivity between nucleus accumbens and left mid-insula. These findings fit with neurological studies showing that strokes affecting the insula can disrupt nicotine addiction,^15, 16^ as well as susceptibility to gambling-related cognitive distortions.^17^ Moreover, meta-analyses of fMRI cue reactivity demonstrate insula recruitment in patients with substance-use disorders,^10^ primarily in more elaborate procedures involving polymodal cues.^49^

To our knowledge, this paper is the first to investigate functional connectivity changes in gambling disorder during a cue reactivity task. Although a reliable group difference was not observed in the ventral striatum in the activity analysis, significant increases were observed in the gambling disorder group in functional connectivity between nucleus accumbens and insula. Furthermore, individual differences were observed in connectivity strength within the gamblers, as a function of cravings and depression. Patients who reported higher levels of craving in response to the gambling cues showed reduced connectivity between the nucleus accumbens and the medial PFC. Reduced activation in the medial PFC has previously been observed in cocaine use disorders during a rewarded cue reactivity task.^50^ Using a cognitive appraisal manipulation in smokers to focus on the short-term or long-term consequences of smoking, Kober *et al.*^22^ reported increases in medial and dorsolateral PFC activity, coupled with decreases in ventral striatal activity and both effects correlated with changes in craving. Disruptions of the prefrontal cortical control over the limbic system, giving rise to disinhibited, impulsive behavior is a central tenet of modern addiction models.^51, 52^ Changes in functional connectivity tied to cravings provide a direct instantiation of such hypotheses in the context of gambling disorder.

Craving was also correlated with length of abstinence in our sample: as abstinence increased, the craving elicited by the gambling cues decreased, replicating previous findings in gambling disorder^53^ (although we note that in substance addictions, this effect is not always observed^54^ or can be non-linear^55^). One interpretation is that length of abstinence is a modulator of neural activity (c.f. ref. 56) and should be examined as such in future studies in gambling disorder. Importantly, overall gambling severity (on PGSI) did not predict either craving or neural activity. Whereas craving and abstinence reflect current clinical state, the PGSI score emphasizes gambling harms (primarily financial consequences) across the last 12 months.

Our results contrast with a previous cue reactivity experiment in gambling disorder showing reduced activity in medial PFC using script-induced imagery.^11^ Some salient methodological differences may account for this discrepancy, including clinical status (community-recruited gamblers in the earlier study versus abstinent gamblers in treatment) and mode of cueing. In neuroimaging studies across the addictions, an increased neural response is typically observed when Pavlovian-conditioned stimuli are presented directly within the task.^57^

As a strength, our experiment included an appetitive control condition in an effort to arbitrate between two psychological theories of addiction. The incentive senitization theory^5^ predicts an increased response in reward-related circuitry to addiction-related cues, but is agnostic in the response to natural rewards.^24, 57^ In contrast, the reward-deficiency hypothesis predicts a generalized decreased response to both addiction-related and natural rewards. Past experiments in addicted groups have supported the reward-deficiency account of the response to natural rewards. This includes a blunted response to the anticipation of erotic imagery in patients with gambling disorder,^27^ to erotic imagery in cocaine dependence^58^ and to food cues in smokers.^28^ Our results are more in line with incentive sensitization. We observed an increased cue reactivity response to gambling stimuli, but this did not transfer to any group differences in response to the appetitive food cues. Nevertheless, this null result must be interpreted with appropriate caution. The cue reactivity network we observed in response to food cues was less extensive than the equivalent response to gambling cues. These responses typically vary by hunger levels, such that fasted participants show an amplified response compared with fed participants.^29, 43^ Our participants ate a small meal 2 h before the MRI scan, and the results may have been affected by this motivational state. This decision to not increase the fasting time before the scan was driven by evidence that gambling-related decision making is modulated by hunger state,^59^ an effect which may have confounded the response to gambling stimuli throughout the fMRI session. In addition, we did not collect food craving ratings during our task, and were therefore unable to look for within-group correlations of the reward network with food craving ratings. Future work might reveal modulations of this network in gambling disorder by scanning participants in a fasted state and obtaining food craving ratings during the task.

Concerning our between-groups analysis, we compared patients with gambling disorder to healthy controls who, although matched on many dimensions, differed significantly on measures of depression, anxiety, alcohol use and smoking, as is typically the case for gambling disorder.^60^ To investigate whether the group differences we observed might actually be driven by these group differences, we entered BDI and AUDIT scores into correlational analyses within the gambling disorder group. There was no overlap in the areas that were activated in our between-group contrasts and regions correlating with BDI or AUDIT scores, suggesting our group differences were not driven by these measures. However to more thoroughly test this, future research should use control participants matched for these common comorbidities. Moreover, future work on gambling cravings would benefit from comparing patients with gambling disorder against regular (but non-problematic) gamblers, to address the more tailored question of why some people can gamble regularly without escalation to problematic gambling, whereas others cannot.^61^ Similarly, it would be fruitful to directly compare cue reactivity in patients with gambling disorder and substance-use disorders using this paradigm,^14^ to characterize the degree of overlap in cravings responses.

In conclusion, the present study characterizes cue-related neural activity in gambling disorder, delineating changes in regional activity and connectivity, as well as the specificity to addiction-related stimuli over natural rewards. In particular, the close relationships between cue-related neural activity in the insula and individual differences in subjective craving may have clinical utility for examining changes over the course of treatment, as previously seen for alcohol use disorders.^62^ Moreover, novel experimental therapeutics such as transcranial magnetic stimulation to deep structures may be capable of reducing insula activity, and are being trialed for treatment of cravings in nicotine dependence.^63^ In formulating the DSM-5 criteria for substance-use disorders, the addition of craving as a diagnostic criteria was motivated less by its leverage in diagnosis, but rather by its value as a useful biomarker for treatment.^1^ Similarities in the neural substrates of cue reactivity between gambling disorder and in substance-use disorders supports the inclusion of craving in future revisions of diagnostic criteria for gambling disorder.

## Figures and Tables

**Table 1:** Group characteristics

	*Gambling disorder*	*Controls*	
Age (years), median (range)	31 (27–51)	28 (25–52)	*W*=217, *P*=0.292, *r*=−0.17
IQ, median (range)	115 (83–134)	118 (78–131)	*W*=166, *P*=0.682, *r*=−0.0664
BDI-II, median (range)	7 (0–41)	0 (0–12)	*W*=323, ***P*****<0.001**, *r*=−0.680
STAI—trait, mean (s.d.)	43.4 (11.7)	33 (10.7)	*t*(35)=2.80, ***P*****<0.01**, *r*=0.429
STAI—state, median (range)	33 (20–77)	25.5 (20–40)	*W*=275, ***P*****<0.01**, *r*=−0.511
FTND, mean (s.d.)	4.57 (1.72)	1.67 (1.97)	*t*(10.1)=2.81, ***P*****<0.05**, *r*=,663
No of smokers (no of ex-smokers)	7 (1)	6 (3)	—
AUDIT, mean (s.d.)	6.74 (3.68)	3.89 (2.47)	*t*(31.5)=2.80, ***P*****<0.01**, *r*=0.446
Hours since meal, mean (s.d.)	2.89 (0.588)	2.73 (0.814)	*t*(29.1)=0.620 *P*=0.5040, *r*=0.114
BMI (kg/m^2^), median (range)	25.1 (20.2–42.2)	23.5 (20.1–28)	*W*=253, *P*<0.05, *r*=−0.341
EDE-Q restraint, median (range)	0 (0–1.8)	0.3 (0–3)	*W*=149, *P*=0.672, *r*=−0.069
DEBQ restraint, median (range)	1.4 (0.9–3.2)	1.5 (1–3.5)	*W*=138*, P*=0.456, *r*=−0.121
TFEQ restraint, median (range)	3 (1–10)	5 (2–18.0)	*W*=105, *P*=0.110*, r*=−0.260

Abbreviations: AUDIT, alcohol use disorders identification test; BDI-II, Beck Depression Inventory; BMI, body mass index; DEBQ, Dutch eating behaviour questionnaire; EDE-Q, eating disorder examination questionnaire; FTND, Fagerstrom test for nicotine dependence; IQ, intelligence quotient; STAI, Spielberger state-trait anxiety index; TFEQ, three factor eating questionnaire.

If data were normally distributed, means and s.d. are shown and unpaired *t*-tests were used to test for group differences.

If data were not normally distributed, medians and ranges are shown and Wilcoxon rank-sum tests were used to test for group differences. Significant group differences are highlighted in bold. Owing to the missing data, one control did not contribute to the STAI analyses, and one control and one gambler did not contribute to the dietary restraint analyses. Hours fasted refers to the time of least meal relative to the start of the cue reactivity task; two participants in each group, tested in the mornings, had not yet eaten on the test day and were omitted from this analysis.
