# Supplementary material for: Neural substrates of cue reactivity and craving in gambling disorder
Source: Transl Psychiatry. 2017 Jan 3;7(1):e992–. doi: 10.1038/tp.2016.256 (PMC5545724; doi:10.1038/tp.2016.256)
Supplement: Supplementary Information [file tp2016256x1.docx]

**Supplementary Information**

**1: Inclusion criteria.**

Participants were considered for inclusion if they were male, between 25 and 60 years of age, and were able to understand English. Additional inclusion criteria for the Gambling Disorder group included: 1) met diagnosis for Pathological Gambling according to the DSM-IV diagnostic criteria; as such cases necessarily meet DSM-5 criteria for Gambling Disorder, we adopt the current term throughout the manuscript for consistency, 2) waiting for, or undergoing, cognitive behavioural therapy for Gambling Disorder and 3) were able to abstain from gambling for 48 hours prior to the study session. The main exclusion criteria for all participants included a current or past history of dependence on substances of abuse (excluding nicotine), use of illegal drugs for two weeks prior to the study, taking psychotropic medication, or having a neurological diagnosis or clinically significant head injury. Participants were excluded if they suffered from a past or current DSM-IV Axis I psychiatric illness, although a past diagnosis of depression or anxiety was allowed for patients with Gambling Disorder since it is common. Participants were tested for illegal drug use and alcohol intoxication using a urine screen and breath alcohol test on the day of the study, and a positive result in either led to exclusion from the study.

**2: Imaging procedure**

High-resolution T1-weighted volumes were acquired using a magnetization-prepared rapid gradient echo (MPRAGE) sequence (TR = 2300 ms, TE = 2.98 ms, TI = 900 ms, flip angle = 9°, field of view = 256 mm, image matrix = 240 x 256) with a resolution of 1 mm isotropic. For the volume, 160 abutting straight sagittal slices were collected in an interleaved right to left manner, resulting in whole head coverage. Parallel imaging using Generalized Autocalibrating Partially Parallel Acquisition (GRAPPA) with an acceleration factor of 2 was performed.

Functional imaging was performed using a multi-echo gradient echo echoplanar imaging (EPI) sequence (TR = 2000 ms, TE = 13 & 31 ms, flip angle = 80°, field of view = 225 mm, image matrix = 64 x 64) with an in-plane resolution of 3.516 x 3.516 mm and a slice thickness of 3.000 mm. The phase encoding direction was anterior to posterior. Echo spacing was 0.52 ms. For each volume, 36 abutting oblique axial slices were collected in an ascending manner at an angle of around 30° to the anterior (AC) and posterior commissure (PC) line. This results in slightly less than whole brain coverage, with the most superior 9 mm not being imaged in most subjects. To achieve the desired resolution and repetition time, parallel imaging using GRAPPA with an acceleration factor of 2 was performed. The first three volumes of each functional run were automatically discarded to allow for T1 saturation effects. Visual stimuli were presented to participants using an MRI compatible screen viewed through a mirror. Responses were collected using a custom made three-button box held in the participants’ dominant hand. E-Prime 2.0 (Psychology Software Tools, Pittsburgh, PA) was used to deliver the task.

**3: Task design.**

Participants viewed a total of six blocks of each cue category. Gambling blocks contained images from the same gambling sub-type. After the rating screen, a fixation cross was presented for at least one second, to ensure the duration of the block was fixed at thirty seconds.

Blocks were organized into sets. A set contained a block from each of the four categories of cues and a rest block. Each run contained three sets. To ensure that the experimental conditions were distributed throughout the runs, the content of each block and each set was fixed, but we randomized the image presentation order within each block, the order of the blocks within each set, and the order of each set within each run.

**4: Additional behavioural analyses.**

To maintain attention to the visual stimuli, participants were instructed to press a button when each new cue was presented. Participants successfully achieved this 87.5% of all cue presentations. A mixed-model ANOVA was used to test for potential differences in the adherence to task instructions between the two groups, and between the Gambling and Neutral blocks. The number of successful button presses was not modulated by Cue type, F(1,36) = 0.30, p = .59, but was modulated by Group, F(1,36) = 5.02, p < .05, with lower rates of responding in the Controls. There was no interaction between these two variables, F(1,36) = 1.75, p = .10. On inspection of the data, three participants from the control group consistently failed to follow the instruction, pressing the button on fewer than 15% of all trials, and accounted for the significant group difference; the remaining Control participants responded at least 79.2% of all trials. These three control participants were retained in the primary analysis. Sensitivity analyses were run with these three control participants removed, and the results were qualitatively unchanged.

For the craving ratings, there was a 5 second window to submit each rating. Overall, ratings were submitted on 95.61% of trials. The number of successfully submitted ratings was not modulated by Gambling cue type, F(1,36) = 0.706, p = .406, Group, F(1,36) = 0.107 , p = .745, or their interaction, F(1,36) = 0.176, p = .677.

**5: fMRI preprocessing**

Pre-processing of the functional data included isolating the second echo scans, standard motion correction (McFLIRT), spatial smoothing (FWHM=7mm) and high pass temporal filtering (120s). T1-weighted structural images were skull stripped in a two step process using MRI-watershed (AFNI) {Cox:1996wd} and BET (FSL). Functional data were registered onto T1 images using boundary based linear registration (FLIRT). Non-linear registration was then used to warp the T1 images onto a 2mm MNI template (10mm warp, FNIRT).

**Supplementary Table 1**

|  |  | (1) | (2) | (3) | (4) | (5) | (6) | (7) | *(8)* | *(9)* | *(10)* | *(11)* | *(12)* |
| --- | --- | --- | --- | --- | --- | --- | --- | --- | --- | --- | --- | --- | --- |
| (1) BDI-II | Pearson’s r | --- | 0.777 | 0.817 | 0.344 | 0.168 | -0.171 | 0.055 | 0.296 | 0.236 | -0.032 | -0.278 | 0.479 |
|  | Sig. (2-tailed) | --- | *** | *** |  |  |  |  |  |  |  |  | * |
| (2) STAI - state | Pearson’s r |  | --- | 0.872 | 0.51 | 0.052 | -0.192 | -0.129 | 0.011 | 0.218 | -0.084 | 0.113 | 0.219 |
|  | Sig. (2-tailed) |  | --- | *** | * |  |  |  |  |  |  |  |  |
| (3) STAI - trait | Pearson’s r |  |  | --- | 0.43 | 0.268 | -0.305 | -0.062 | 0.152 | 0.262 | -0.051 | 0.043 | 0.196 |
|  | Sig. (2-tailed) |  |  | --- |  |  |  |  |  |  |  |  |  |
| (4) PGSI | Pearson’s r |  |  |  | --- | 0.434 | -0.34 | -0.171 | 0.441 | 0.437 | 0.221 | 0.196 | 0.361 |
|  | Sig. (2-tailed) |  |  |  | --- | . | . | . |  |  |  |  |  |
| (5) Craving | Pearson’s r |  |  |  |  | --- | -0.533 | -0.098 | 0.622 | 0.323 | 0.284 | -0.049 | 0.252 |
|  | Sig. (2-tailed) |  |  |  |  | --- | * |  | ** |  |  |  |  |
| (6) Abstinence | Pearson’s r |  |  |  |  |  | --- | 0.119 | -0.565 | -0.388 | -0.493 | -0.178 | -0.095 |
|  | Sig. (2-tailed) |  |  |  |  |  | --- |  | * |  | * |  |  |
| (7) AUDIT | Pearson’s r |  |  |  |  |  |  | --- | 0.046 | -0.055 | -0.108 | -0.486 | -0.159 |
|  | Sig. (2-tailed) |  |  |  |  |  |  | --- |  |  |  | * |  |
| *(8) UPPS-P*  *Negative Urgency* | *Pearson’s r* |  |  |  |  |  |  |  | *---* | *0.608* | *0.562* | *-0.071* | *0.461* |
|  | *Sig. (2-tailed)* |  |  |  |  |  |  |  | *---* | **** | *** |  | *** |
| *(9) UPPS-P*  *Positive urgency* | *Pearson’s r* |  |  |  |  |  |  |  |  | *---* | *0.423* | *-0.061* | *0.489* |
|  | *Sig. (2-tailed)* |  |  |  |  |  |  |  |  | *---* |  |  | *** |
| *(10) UPPS-P*  *Lack of planning* | *Pearson’s r* |  |  |  |  |  |  |  |  |  | *---* | *0.457* | *0.276* |
|  | *Sig. (2-tailed)* |  |  |  |  |  |  |  |  |  | *---* | *** |  |
| *(11) UPPS-P*  *Lack of perseverence* | *Pearson’s r* |  |  |  |  |  |  |  |  |  |  | *---* | *-0.314* |
|  | *Sig. (2-tailed)* |  |  |  |  |  |  |  |  |  |  | *---* |  |
| *(12) UPPS-P*  *Sensation seeking* | *Pearson’s r* |  |  |  |  |  |  |  |  |  |  |  | *---* |
|  | *Sig. (2-tailed)* |  |  |  |  |  |  |  |  |  |  |  | *---* |

Correlation coefficients for the clinical measures *(and impulsivity)* within the Gambling Disorder group. N = 19. BDI-II = Beck Depression Inventory, STAI = Spielberger State-Trait Anxiety Inventory, PGSI = Problem Gambling Severity Index, AUDIT = Alcohol Use Disorders Identification Test, UPPS-P = UPPS-P Impulsive Behaviour Scale, * = p < .05, ** = p < .01, *** = p < .001. Impulsivity measures were not central to our hypotheses or interpretation and so are presented for information only.

**Supplementary Figure 1**

**
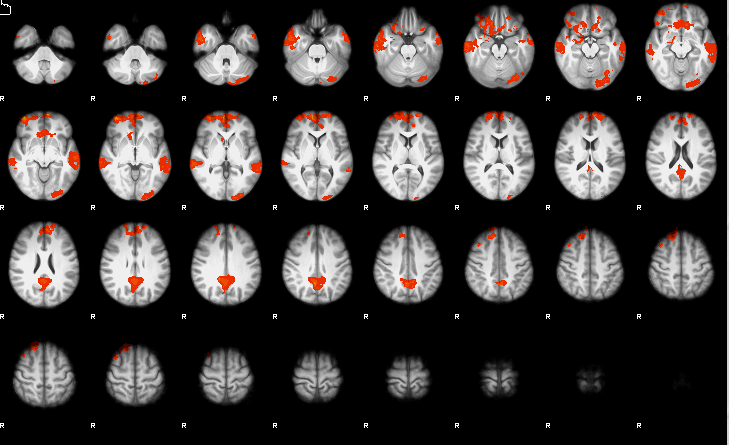
**

Activity resulting from the Gambling cue > Gambling-matched neutral cue contrast in control group. Four clusters of activity were revealed. One peaked within the frontal pole [40, 60, -8, Z = 5.66], one within the precuneus [4, -54, 36, Z = 4.78], one within the middle temporal gyrus [-60, -20, -8, Z = 4.10], and one within the occipital pole [-18, -100, 0, Z = 3.93].­­

**Supplementary Figure 2**


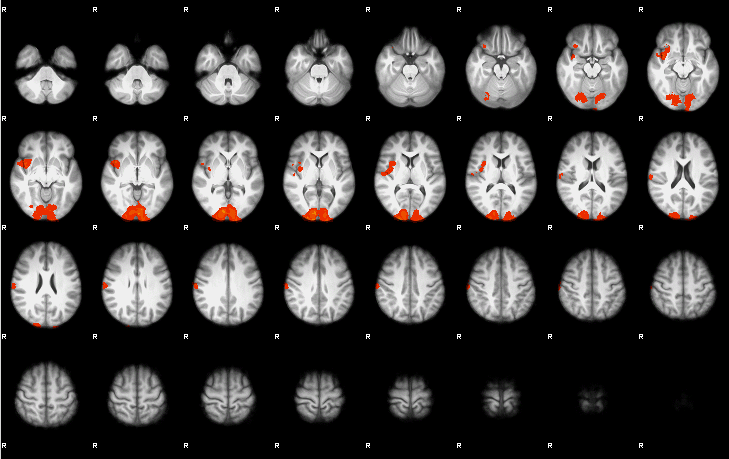


Activity resulting from the Food cue > Food-matched neutral cue contrast in the Gambling Disorder group.

**Supplementary Figure 3**


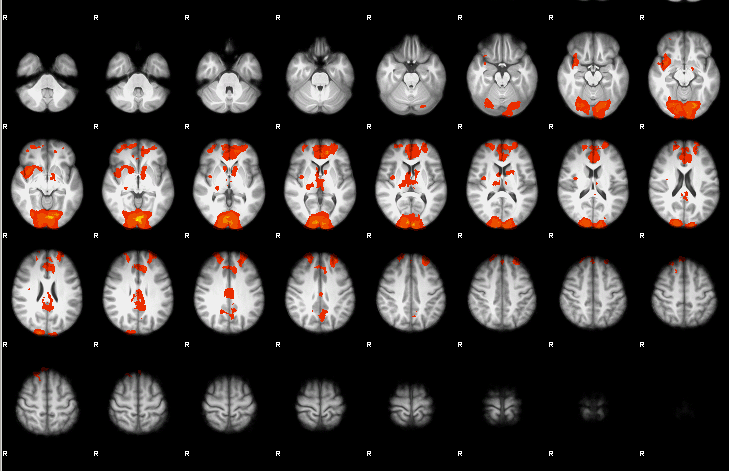


Activity from the Food cue > Food-matched neutral cue contrast when all participants’ data (Gambling Disorder and control groups) were combined.

**Supplementary Figure 4**


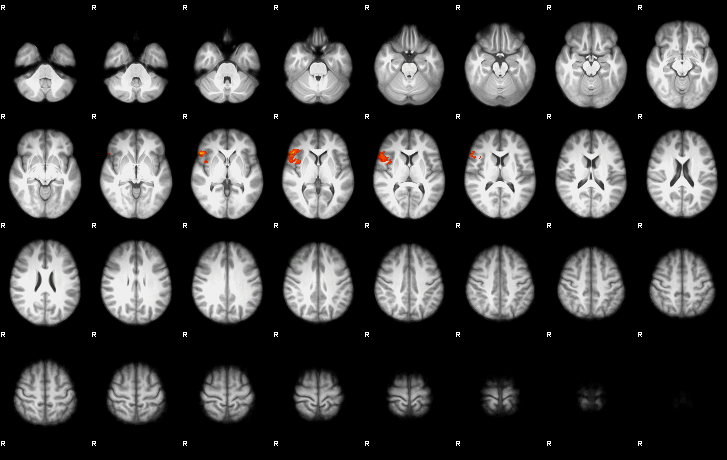


Connectivity results from the Gamble cue > Gambling-matched neutral cue contrast in the Gambling Disorder group.
